# Postprandial Metabolic Response to Rapeseed Protein in Healthy Subjects

**DOI:** 10.3390/nu12082270

**Published:** 2020-07-29

**Authors:** Christin Volk, Corinna Brandsch, Ulf Schlegelmilch, Monika Wensch-Dorendorf, Frank Hirche, Andreas Simm, Osama Gargum, Claudia Wiacek, Peggy G. Braun, Johannes F. Kopp, Tanja Schwerdtle, Hendrik Treede, Gabriele I. Stangl

**Affiliations:** 1 Institute of Agricultural and Nutritional Sciences, Martin Luther University Halle-Wittenberg, 06120 Halle, Germany; christin.volk@landw.uni-halle.de (C.V.); corinna.brandsch@landw.uni-halle.de (C.B.); ulf.schlegelmilch@landw.uni-halle.de (U.S.); monika.dorendorf@landw.uni-halle.de (M.W.-D.); frank.hirche@landw.uni-halle.de (F.H.); 2 Department of Cardiac Surgery, University Hospital Halle (Saale), Martin Luther University Halle-Wittenberg, 06120 Halle, Germany; andreas.simm@uk-halle.de (A.S.); osama.gargum@uk-halle.de (O.G.); hendrik.treede@ukbonn.de (H.T.); 3 Institute of Food Hygiene, University Leipzig, 04103 Leipzig, Germany; claudia.wiacek@vetmed.uni-leipzig.de (C.W.); pbraun@vetmed.uni-leipzig.de (P.G.B.); 4 Institute of Nutritional Science, University of Potsdam, 14558 Nuthetal, Germany; jokopp@uni-potsdam.de (J.F.K.); taschwer@uni-potsdam.de (T.S.); 5 Department of Cardiac Surgery, Heart Center Bonn, University Hospital Bonn, 53127 Bonn, Germany

**Keywords:** rapeseed protein, soy protein, postprandial study, metabolic response, healthy subjects

## Abstract

Plant proteins have become increasingly important for ecological reasons. Rapeseed is a novel source of plant proteins with high biological value, but its metabolic impact in humans is largely unknown. A randomized, controlled intervention study including 20 healthy subjects was conducted in a crossover design. All participants received a test meal without additional protein or with 28 g of rapeseed protein isolate or soy protein isolate (control). Venous blood samples were collected over a 360-min period to analyze metabolites; satiety was assessed using a visual analog scale. Postprandial levels of lipids, urea, and amino acids increased following the intake of both protein isolates. The postprandial insulin response was lower after consumption of the rapeseed protein than after intake of the soy protein (*p* < 0.05), whereas the postmeal responses of glucose, lipids, interleukin-6, minerals, and urea were comparable between the two protein isolates. Interestingly, the rapeseed protein exerted stronger effects on postprandial satiety than the soy protein (*p* < 0.05). The postmeal metabolism following rapeseed protein intake is comparable with that of soy protein. The favorable effect of rapeseed protein on postprandial insulin and satiety makes it a valuable plant protein for human nutrition.

## 1. Introduction

The substitution of animal proteins with plant proteins has become increasingly important in human nutrition for ecological and health reasons. Ecologists recommend limiting the intake of proteins from animal sources to reduce greenhouse gas emissions and the use of biological resources [1]. Nutritionists and clinicians emphasize the beneficial effects of dietary plant proteins versus animal proteins on lipid and glucose metabolism and for the prevention of obesity and hypertension [2,3,4,5,6,7].

Among protein crops, soy and other legumes, such as lupin, are characterized by a favorable profile of indispensable amino acids and are thus an important component of the human diet. Another promising plant source of protein is rapeseed. Rapeseed is primarily used for the production of edible rapeseed oil. Protein-rich by-products from oil production, such as rapeseed cake or meal, are predominantly used as animal feed. Analyses have revealed that the amino acid composition of rapeseed proteins is comparable to that of other legume proteins [8]. This indicates that rapeseed protein, in principle, meets the requirements for being an indispensable amino acid source for human consumption [9]. Fleddermann et al. were the first to demonstrate that the intake of rapeseed protein resulted in a postprandial amino acid profile that was comparable to that of soy protein in healthy male volunteers [10]. Both the amino acid composition and the data on postprandial amino acid levels in humans demonstrate that rapeseed protein has the potential to become a valuable dietary compound. In contrast to soy protein, rapeseed protein does not contain isoflavones, which have been rated as critical due to their estrogenic activity [11]. In addition, as a source of amino acids, plant proteins are considered to have additional health effects. The majority of human studies that have explored the health benefits of plant proteins were conducted with soy protein. Data from these studies demonstrated beneficial effects of soy protein on glucose metabolism, plasma cholesterol, and inflammatory markers in patients with type 2 diabetes compared to either animal protein or no additional protein [3,12,13,14,15]. As a consequence, in 1999, the US Food and Drug Administration (FDA) authorized the use of a health claim for soy-based foods to tout their heart-healthy benefits, such as their cholesterol-lowering potential, and recommended a total of 25 g of soy protein to achieve this health [16].

Plant protein isolates, in contrast to animal proteins, can contain phytochemicals, which may also impact health. Rapeseed is characterized by high quantities of natural antioxidants such as tocopherols and sinapic acid derivatives, which belong to the phenol acids [17,18,19]. Rapeseed oil also contains significant amounts of phytosterols, which are suggested to have a beneficial impact on cholesterol levels [20]. Other relevant phytochemicals in rapeseed protein are glucosinolates and phytic acid. Glucosinolates are well-described anti-nutritive factors that can exert thyreostatic effects [21], but they are also suggested to have preventive effects on cancer and neurodegenerative diseases [22,23]. Rapeseed varieties that are used in human nutrition normally contain low levels of glucosinolates. In contrast to glucosinolates, phytic acid exerts its anti-nutritive effects by impairing the intestinal absorption of minerals, particularly zinc [24]. It was shown that the reduction of phytic acid in food can improve the availability of minerals [24,25]. Apart from that, plant protein isolates are also valuable sources of minerals [26].

A third aspect of dietary proteins is their influence on satiety and subsequent energy intake. It is widely known that the intake of high-protein diets induces greater satiety than that of low-protein diets [27,28]. In addition to the quantity of ingested protein, various protein sources appear to have differential effects on satiety [29].

While soy protein effects have been intensively studied in humans, there are virtually no data concerning the health impacts of rapeseed protein. One approach to evaluate the beneficial or detrimental effects of a food component on health is to study the postprandial metabolic response. Since postprandial levels of plasma glucose [30,31] and lipids [32,33,34] are independent risk factors for cardiovascular diseases, studies elucidating the impact of dietary factors on levels of postprandial metabolites are highly relevant in evaluating the health impacts of these diets.

To shed more light on the postprandial effects of rapeseed protein, we conducted a randomized controlled trial with healthy subjects in which we compared the effects of rapeseed with soy protein or no additional protein on postprandial glucose levels, which was the primary study outcome. Besides plasma glucose, we investigated the postprandial response of metabolites and cardiovascular risk factors, satiety and postmeal appetite, and postprandial levels of minerals and hormones involved in the regulation of mineral homeostasis. The findings from this study may serve as a basis to comprehensively evaluate the suitability of rapeseed protein for use in human nutrition.

## 2. Materials and Methods

### 2.1. Study Design and Study Population

This study was conducted as a double-blind, randomized, controlled human intervention trial with a crossover design. All subjects gave their informed consent for inclusion before they participated in the study. The study was conducted in accordance with the Declaration of Helsinki, and the protocol was approved by the Ethics Committee of the Medical Faculty at Martin Luther University Halle-Wittenberg (2018-50, date of approval: 12 June 2018). The study was registered at clinicaltrials.gov (NCT03620812).

Healthy subjects were recruited through advertisements on the university intranet, personal contacts, and public information events between June and August 2018. Subjects were apparently healthy, aged between 18 and 65 years, had body mass indexes (BMIs) between 18.5 and 30.0 kg/m^2^ and were nonsmokers to meet the inclusion criteria. Subjects not included were those with acute or chronic diseases, allergies or intolerances to soy, tomato, wheat, or mustard, those who received prescription medication, were pregnant or nursing, were following a diet, or had donated blood during the last two months. Subjects also not included were those who participated in other clinical studies and who were very physically active. All volunteers had to fill out a questionnaire on their medical history, lifestyle behaviors (e.g., smoking, physical activity), allergies, body weight, and height.

The primary parameter for sample size calculation was postprandial plasma glucose. Sample size was calculated using a Java applet [35] to determine statistically significant differences in the plasma glucose response (0–180 min area under the curve, AUC) between the three treatments. Using a balanced ANOVA for crossover design as a statistical test, a mean difference of 35% between the treatments with additional protein and the treatment without additional protein [36], a power of 90%, and a significance level of 0.05, a total sample size of 18 subjects was found to be required [28] (Java Applets for Power and Sample Size [Computer software]; Retrieved February 20, 2018, from http://www.stat.uiowa.edu/~rlenth/Power). The dropout rate was estimated to be 30%. Thus, a total of 24 subjects were involved in the study.

The intervention assumed that each subject received one of the following dietary treatments: standardized test meal without additional protein (TM), standardized test meal with 28 g of rapeseed protein isolate (TM+RPI), or standardized test meal with 28 g of soy protein isolate (TM+SPI). The quantity of administered protein isolate was calculated to achieve an actual additional intake of 25 g of pure protein. The rationale for using 25 g of pure protein was the soy health claim that considers 25 g soy protein per day as clinically relevant [16]. To avoid time-dependent treatment effects, the study participants were randomly assigned to one of the six possible sequences (Figure S1) by block randomization using a computer-generated randomization schedule with sex as the stratification criteria. The wash-out period between the dietary treatments was set to be at least two weeks but not more than seven weeks. Four participants dropped out for personal reasons. Thus, 20 participants completed the study.

The participants were asked to fill out a 3-day dietary protocol in the week prior to the first study day to analyze their usual dietary habits via the DGExpert program (DGExpert 1.7). Prior to each intervention, subjects underwent a three-day run-in period in which they were not allowed to drink alcohol or be physically active. The evening before the day of intervention, the participants received a standardized meal (bread with cheese and butter, an apple, and yogurt; 743 kcal, 88 g carbohydrates, 31 g fat, and 25 g protein) that was consumed no later than 19:00 to minimize the effects of the last meal on the postprandial response.

**Figure 1:**
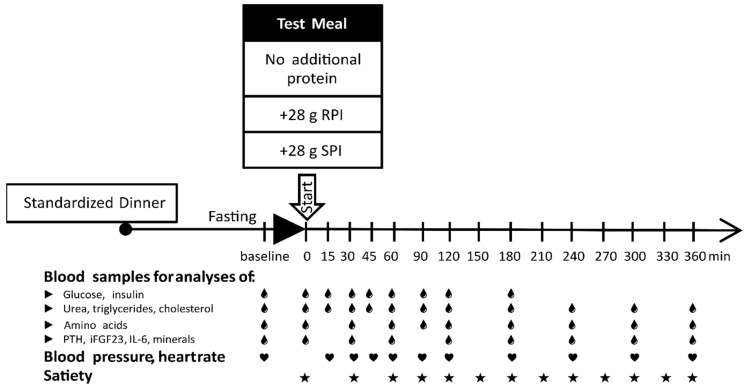
Study schema outlining the dietary interventions and the time points of blood sampling for intended analyses (droplets), blood pressure, and heart rate (hearts) measurements and assessment of satiety (asterisks). Test meals (pasta with tomato sauce and corn oil) were administered without additional protein or with 28 g of either rapeseed protein isolate (RPI) or soy protein isolate (SPI). The meals were served at 8.00 a.m.

After overnight fasting for at least 12 h, the participants visited the University Hospital of Martin Luther University in Halle. All individuals rested at least 5 min prior to measurement of their blood pressure and heart rate in triplicate at the dominant arm with a one-minute interval in between measurements (BpTRU Medical Devices, Coquitlam, BC, Canada). Then, an indwelling intravenous cannula was inserted into the participants (superficial arm veins) for blood sampling at the nondominant arm. Prior to consumption of the test meals, the first venous blood sample was taken for analyses of the fasting plasma parameters (baseline). Then, the participants were asked to eat the standardized test meal within 10 min. Immediately after consumption of the test meal, the next blood sample was taken (0 min), and then samples were taken at 15, 30, 45, 60, 90, 120, 180, 240, 300, and 360 min afterwards (Figure 1). Blood pressure and heart rate were also determined at the aforementioned time points. Except for water, participants were not allowed to consume any food during the 6-h postprandial trial period. Every 30 min during these 6-h periods, the participants were asked to fill out a visual analog scale to assess their postprandial feelings of appetite and satiety.

### 2.2. Preparation and Composition of the Meals

The test meal consisted of 120 g boiled wheat pasta (cooking time 12 min), 150 g tomato sauce, and 20 g corn oil. The test meal was supplemented with no additional protein, 28 g rapeseed protein isolate, or 28 g soy protein isolate, which was used as control protein. Rapeseed or soy protein isolate was added to the tomato sauce and cooked for 1 min before being added to the pasta. By mixing the protein isolates in the tomato sauce, the different tastes of the protein isolates were masked. This assured the blinding of the intervention. The nutrient composition of the three test meals is shown in Table 1.

### 2.3. Preparation and Composition of the Proteins

The rapeseed (*Brassica napus*; Cultivar Visby) protein isolate was prepared by Pilot Pflanzenöltechnologie Magdeburg e.V. (Magdeburg, Germany). It consisted of napin (62.4%) and cruciferin (36.2%), which are the two major storage proteins in rapeseed. The napin–cruciferin ratio resulted from the isolation process and did not necessarily represent the natural proportions, although the usual napin–cruciferin ratio can show great variation in the range between 0.6 and 2.0 [37]. For protein isolation, the rapeseed was thermally treated (80 °C), and its oil was extracted with n-hexane. Subsequently, the rapeseed cake was further processed by separation, ultrafiltration, and spray drying. An ultrahigh temperature-treated soy protein isolate Dunapro95M was obtained from Euroduna Food Ingredients GmbH (Barmstedt, Germany).

The protein samples were analyzed for six microbiological parameters (the total aerobic bacterial count (DIN EN ISO 4833-2, 2014-04), yeast and mold (ASU L01.00-37 1991-12), *Enterobacteriaceae* (DIN EN ISO 10164-1, 2019-06), *Salmonellae* (DIN EN ISO 6579-1, 2017-07), *Listeria monocytogenes* (DIN EN ISO 11290-1, 2017-09), *Bacillus cereus* (DIN EN ISO 7932, 2004-03, Amendment: use of PEMBA instead of MYP agar), and coagulate positive *staphylococci* (DIN EN ISO 6888-1, 2019-6). All microorganisms analyzed in the soy protein isolate were markedly below the standard and warning values of the German Society for Hygiene and Microbiology (for oil seeds) for these microorganisms. To reduce the aerobic colony counts in the rapeseed protein and to ensure a microbiological quality comparable to that of soy protein, the rapeseed protein isolate was thermally treated for 60 s at 135 °C.

The dry matter, crude nutrient content, and amino acid composition of the experimental protein isolates (Table 2) as well as their quantities of sodium, potassium, calcium, and phosphorus (Table 3) were analyzed by a local laboratory (CBA GmbH, Böhlen, Germany) according to official methods (VDLUFA 3.1; 1976; 4.11.1, 1997; 4.11.2, 1988; 10.8.2; 2006). Zinc and copper (Table 3) were analyzed by ICP-MS/MS. Therefore, samples were pretreated with HNO_3_ (65% suprapur) and H_2_O_2_ (30% suprapur) and microwave digested (200 °C, 20 min) [38]. The crude fat and crude fiber levels of the protein isolates were analyzed using official methods [39,40]. The crude ash content was ascertained by incineration in a muffle furnace at 550 °C.

The concentration of phytate in the proteins was measured according to the method of Harland and Oberleas [41]. In brief, dried samples of proteins were extracted with 2.4% HCl. Then, the extracts were filtered and subjected to a chromatographic system (column: AG1-X4, Bio-Rad Laboratories, Hercules, CA, USA). After the column was washed with 0.1 M NaCl, the phytate was eluted with 0.7 M NaCl. The phytate-containing fractions were wet ashed with 1 M H_2_SO_4_ and 5 M HClO_4_ at 250 °C, and the inorganic phosphate was analyzed colorimetrically using standards. The phytic acid content of the sample was calculated to be 28.2% × phosphorus.

### 2.4. Satiety and Appetite Assessment

The participants were asked to record their appetite sensation every 30 min over 6 h postprandial by using visual analog scales (VAS). Four 100-mm scales were established for the following statements: (1) hunger (“I do not feel hungry” = 0 mm to “very hungry” = 100 mm), (2) satiety (“not satisfied” = 0 mm to “very satisfied” = 100 mm), (3) fullness (“not full” = 0 mm to “completely full” = 100 mm), and (4) prospective consumption (“I don’t want to eat my favorite dessert” = 0 mm to “I want to eat my favorite dessert” = 100 mm).

### 2.5. Blood Sampling and Analysis

For analyses of metabolites, hormones, and minerals, venous blood samples were collected in heparinized tubes (Sarstedt, Nümbrecht, Germany). The samples were centrifuged at 2500× *g* for 15 min to obtain plasma. For the quantification of glucose, fluoride-coated tubes (Sarstedt) were used, and plasma was separated no later than 1 h after drawing. Plasma samples were aliquoted and stored at −80 °C until analyses.

Plasma concentrations of glucose, triglycerides, cholesterol, and urea were measured using enzymatic assays (DiaSys Diagnostic Systems GmbH, Holzheim, Germany). Plasma levels of inorganic phosphate and calcium were determined using spectrophotometric assays (Analyticon Biotechnologies AG, Lichtenfels, Germany). Plasma concentrations of interleukin-6 (IL-6) (IBL International GmbH, Hamburg, Germany), insulin (DRG Instruments GmbH, Marburg, Germany), parathyroid hormone (PTH) (Immutopics Inc, San Clemente, CA, USA), intact fibroblast growth factor-23 (iFGF23) (Immutopics Inc), and high-sensitivity C-reactive protein (hs-CRP) (IBL International GmbH) were analyzed by the use of commercially available ELISAs according to manufacturer’s protocol. Circulating zinc and copper were analyzed by ICP-MS/MS as previously described [42]. In brief, 50 µL of plasma sample were diluted 1 + 9 with an alkaline solution (ammonia, EDTA, butanol and water) and directly analyzed via ICP-MS/MS (Agilent 8800, Agilent Technologies, Waldbronn, Germany). The accuracy of the results was confirmed using ClinChek^®^ lyophilized control serum (Ref. 8880-8882, LOT 1497, RECIPE Chemicals + Instruments GmbH, Munich, Germany).

The concentrations of amino acids in plasma were determined as isoindole derivatives by reversed-phase HPLC (HPH C18, 100 mm × 4.6 mm, 2.7 µm, Agilent 1260 Infinity II, Agilent Technologies) after precolumn derivatization with o-phthaldialdehyde and mercaptopropionic acid [43,44]. The amino acid concentrations were quantified relative to a calibrated plasma (ClinCal^®^ calibrator, RECIPE Chemicals + Instruments GmbH).

### 2.6. Statistical Analysis

Statistical analysis was performed using the SAS 9.4 software package (SAS Institute Inc., Cary, NC, USA). Least square means (LSM) ± standard error (SE) were estimated using the SAS MIXED procedure. The incremental area under the curve (iAUC) was calculated for each subject and treatment using the trapezoidal rule. The mixed-models procedure (PROC MIXED) was used for all traits. For iAUC traits, treatment, gender, the sequence (order) of treatments, and the period (time, when the experimental diets were given) were set as fixed effects, and the subject was included as a random effect. The traits that were repeatedly recorded within a time interval were evaluated with a mixed model, according to the three-period crossover trial model with repeated measurements [45], to test the effects of treatment, time, and their interaction (treatment × time) on each parameter. The value at baseline (before treatment) was considered a covariate. The model of Jones and Kenward [45], which we used for statistical analysis, was extended to include the effect of sex. If convergence could not be achieved, a simpler residual covariance structure such as Banded Toeplitz with three bands was modeled instead of an unstructured covariance. All *p* values were adjusted according to the Tukey–Kramer multiple group comparison procedure. For time point analysis, the paired t-test was applied. Outliers were defined according to the three sigma rule. The significance level was set at 5%.

## 3. Results

### 3.1. Characteristics of the Test Proteins

Both protein isolates were characterized by high concentrations of crude protein > 90%. The remaining components were fat, fiber, minerals, and phytic acid, which was found at a concentration of 2% in both isolates analyzed. According to the manufacturer’s information, the soy protein isolate contained 0.17 g isoflavones per 100 g dry matter (DM). The differences in the amino acid composition between the two isolates were rather small. Among the indispensable amino acids, the rapeseed protein isolate contained higher quantities of the sulfur-containing amino acids methionine and cysteine and moderately lower quantities of the branched-chain amino acids lysine and phenylalanine. The most marked differences in nonprotein components between the two isolates were observed for calcium, which was markedly higher in the rapeseed protein isolate than in the soy protein isolate.

### 3.2. Subjects

The baseline characteristics of the study participants are presented in Table 4. The participants were aged between 18 and 64 years (mean age: 31 years), and 75% of them were females. The majority of the participants (75%) had BMI values within the normal range (> 18.5 kg/m^2^ and < 24.9 kg/m^2^), and 25% had BMI values in the overweight range (25–29.9 kg/m^2^). Eighteen participants were normotensive, and two had systolic blood pressure levels between 140 and 159 mmHg and diastolic blood pressure levels between 90 and 99 mmHg. Heart rate and fasting plasma concentrations of glucose, insulin, triglycerides, cholesterol, hs-CRP, IL-6, iFGF-23, PTH, inorganic phosphorus, calcium, zinc, copper, and urea were within normal ranges. One participant was hyperglycemic according to the American Diabetes Association. This participant was excluded from the statistical analyses of plasma glucose and insulin. One participant did not complete the last 3 h of the last intervention day. Data from this participant were used for the statistical analyses of plasma glucose and insulin but not for parameters analyzed over the 6 h postprandial period. The baseline amino acid concentrations of the study participants are presented in Table S1.

Evaluation of the food records of the participants showed that the mean daily energy intake was 2031 kcal (2031 ± 419 kcal; 1240–3099 kcal), and the mean protein intake was 1.11 g/kg body weight (1.11 ± 0.07 g/kg; 0.59–1.69 g/kg). Of the total energy consumed, 51% (51 ± 6%; 38–60%) were derived from carbohydrates, and 36% (36 ± 6%; 27–46%) were derived from fat. Three participants were vegetarians and 17 were mixed-food consumers. Self-reported physical activity ranged between 0 and 8 h/week (mean: 1.5 h/week).

### 3.3. Postprandial Heart Rate and Blood Pressure

Postprandial blood pressure was not differentially influenced by the treatments (Figure S2A,B). Treatment with TM+SPI resulted in a marginally higher postprandial heart rate than treatment with TM+RPI (Figure S2C).

### 3.4. Postprandial Satiety and Appetite

**Figure 2:**
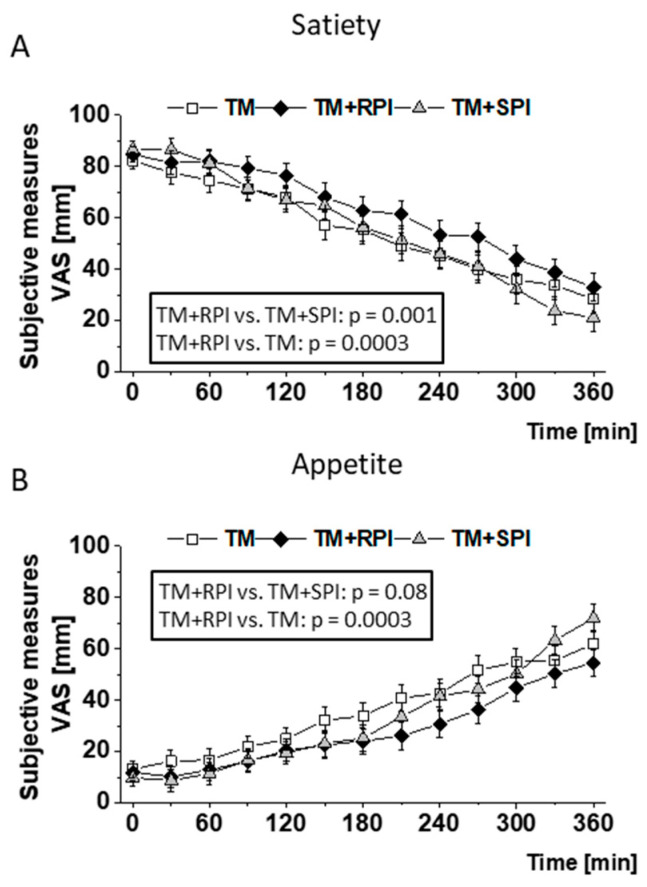
Postprandial satiety (**A**) and appetite (**B**) of study participants who received either the test meal (TM) without additional protein (□), the TM with 28 g rapeseed protein isolate (RPI) (♦), or the TM with 28 g soy protein isolate (SPI) (▲). Satiety and appetite were assessed subjectively by using visual analog scales (VAS) in 30-min intervals. Differences in self-reported postprandial satiety and appetite were evaluated with a mixed model to test the effects of treatment, time, and their interaction (treatment × time) on each parameter. The value at baseline (before treatment) was considered a covariate. All *p* values were adjusted according to the Tukey–Kramer multiple group comparison procedure. For time-point analysis, the paired t-test was applied. Significance was accepted as *p* < 0.05. Data are presented as LSMs ± SEs (*n* = 19).

Evaluation of the VAS demonstrated a better feeling of satiety and a reduced feeling of appetite after the ingestion of TM+RPI than after consumption of TM and TM+SPI, respectively (Figure 2A,B). No differences in satiety and appetite were observed between treatment with TM+SPI and TM, respectively.

### 3.5. Postprandial Plasma Glucose and Insulin

**Figure 3:**
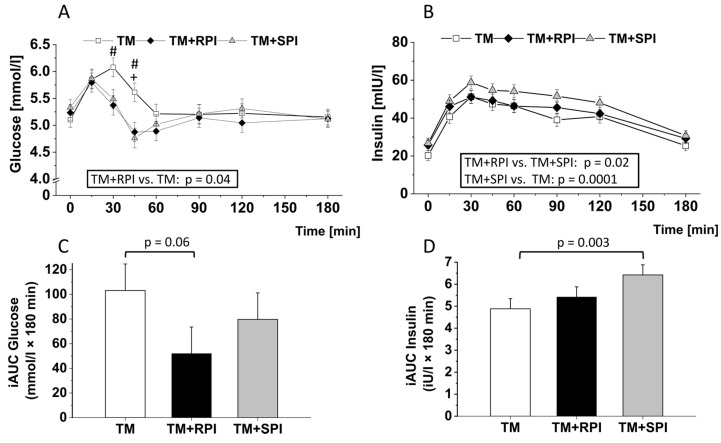
Postprandial response to the test meal (TM) with either no additional protein as reference (□), 28 g rapeseed protein isolate (RPI) (♦), or 28 g soy protein isolate (SPI) (▲) with regard to plasma levels of glucose (**A**) and insulin (**B**) and on the incremental area under the curve (iAUC) of glucose (**C**) and insulin (**D**). Differences in postprandial glucose and insulin concentrations after the ingestion of the three test meals over 3 h were evaluated with a mixed model to test the effects of treatment, time, and their interaction (treatment × time). The value at baseline (before treatment) was considered a covariate. The iAUCs were calculated for each subject and treatment using the trapezoidal rule. The mixed-models procedure (PROC MIXED) was used for all traits. For iAUC treatment, the sex, sequence, and period were set as fixed effects, and subjects were included as random effects. All *p* values were adjusted according to the Tukey–Kramer multiple group comparison procedure. For time-point analysis, the paired t-test was applied. Significance was accepted as *p* < 0.05. Data are presented as LSMs ± SEs (*n* = 19). #, *p* < 0.05 TM vs. TM+RPI; +, *p* < 0.05 TM vs. TM+SPI.

Figure 3A illustrates the postprandial increase in plasma glucose that peaked at 15 min after intake of TM+RPI and TM+SPI, respectively, and at 30 min after the consumption of TM. Thereafter, all treatment groups showed a decline in glucose levels to baseline and below within 60 min postprandial. Statistical analysis of all repeated measurements revealed a significant difference between TM+RPI and TM. Treatment with TM+RPI resulted in significantly lower levels of postprandial glucose than TM. This difference was also seen as a trend by the iAUC of glucose, which was calculated over 3 h (*p* = 0.06, Figure 3C). The iAUC of glucose did not show differences between TM+RPI and TM+SPI or between TM+SPI and TM (Figure 3C).

Postprandial insulin levels are shown in Figure 3B. All dietary treatments caused an increase in the circulating insulin concentrations, which peaked at 30 min postprandial and dropped near baseline 3 h postprandial. Analysis revealed a significantly higher postprandial insulin response after the consumption of TM+SPI than after the consumption of TM and TM+RPI. The increased postprandial insulin response to TM+SPI was also reflected by the significantly higher iAUC calculated for this treatment (Figure 3D). Postprandial insulin concentrations and iAUC of insulin did not show differences between the TM+RPI and TM treatments (Figure 3D).

### 3.6. Postprandial Lipids and Inflammatory Markers

**Figure 4:**
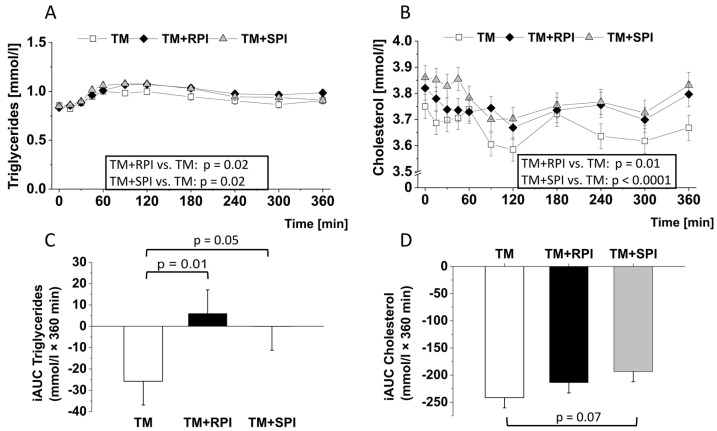
Postprandial response to the test meal (TM) with either no additional protein as reference (□), 28 g rapeseed protein isolate (RPI) (♦), or 28 g soy protein isolate (SPI) (▲) with regard to plasma levels of triglycerides (**A**) and cholesterol (**B**) and the incremental area under the curve (iAUC) of triglycerides (**C**) and cholesterol (**D**). Differences in postprandial glucose and insulin concentrations after the ingestion of the three test meals over 6 h were evaluated with a mixed model to test the effects of treatment, time, and their interaction (treatment × time). The value at baseline (before treatment) was considered a covariate. The iAUCs were calculated for each subject and treatment using the trapezoidal rule. The mixed-models procedure (PROC MIXED) was used for all traits. For iAUC treatment, the sex, sequence, and period were set as fixed effects, and subjects were included as random effects. All *p* values were adjusted according to the Tukey–Kramer multiple group comparison procedure. For time-point analysis, the paired t-test was applied. Significance was accepted as *p* < 0.05. Data are presented as LSMs ± SEs (*n* = 19).

To elucidate the impact of dietary proteins on the postprandial response of lipids, we analyzed plasma levels of triglycerides and cholesterols over a period of 360 min postprandial. Figure 4A illustrates significant differences in the postprandial triglycerides between TM+RPI and TM and between TM+SPI and TM. TM+RPI as well as TM+SPI resulted in moderately higher postprandial triglyceride levels than TM (Figure 4A). No differences in postprandial triglyceride responses were observed between TM+RPI and TM+SPI (Figure 4A). The iAUC of triglycerides was higher after the consumption of TM+RPI and TM+SPI than after the intake of TM (Figure 4C).

All treatments induced a decline in postprandial levels of cholesterol (Figure 4B). In comparison to TM, the decline in postprandial cholesterol was less pronounced after the consumption of TM+SPI or TM+RPI. The negative iAUC values of cholesterol tended to be smaller with TM+SPI than TM (Figure 4D).

**Figure 5:**
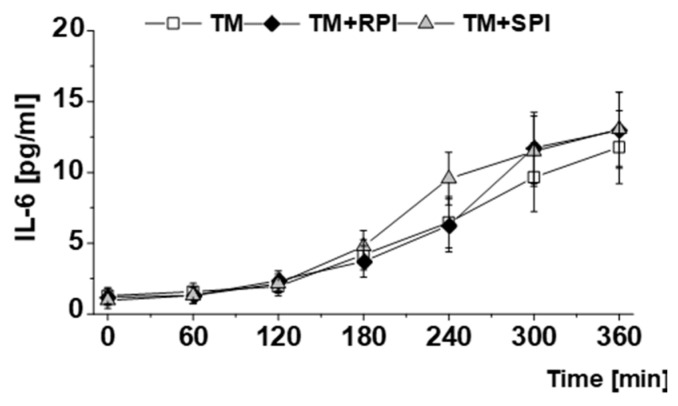
Postprandial response to the test meal (TM) with either no additional protein as reference (□), 28 g rapeseed protein isolate (RPI) (♦), or 28 g soy protein isolate (SPI) (▲) with regard to plasma levels of interleukin-6 (IL-6). Differences in IL-6 concentrations after the ingestion of the three test meals over 6 h were evaluated with a mixed model to test the effects of treatment, time, and their interaction (treatment × time). The value at baseline (before treatment) was considered a covariate. All *p* values were adjusted according to the Tukey–Kramer multiple group comparison procedure. For time-point analysis, the paired t-test was applied. Significance was accepted as *p* < 0.05. Data are presented as LSMs ± SEs (*n* = 19).

The data show a marked increase in the postprandial concentrations of IL-6, which was not influenced by the proteins (Figure 5).

### 3.7. Postprandial Minerals and Mineral-Regulating Hormones

**Figure 6:**
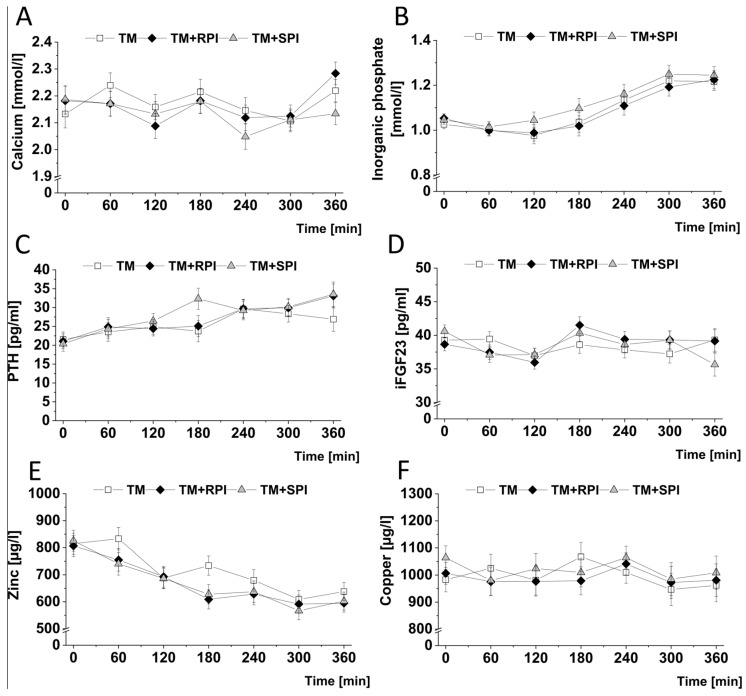
Postprandial response of the test meal (TM) with either no additional protein as reference (□), 28 g rapeseed protein isolate (RPI) (♦), or 28 g soy protein isolate (SPI) (▲) with regard to plasma calcium (**A**), inorganic phosphate (**B**), PTH (**C**), iFGF23 (**D**), zinc (**E**), and copper (**F**). Differences in mineral and related hormone concentrations after the ingestion of the three test meals over 6 h were evaluated with a mixed model to test the effects of treatment, time, and their interaction (treatment × time) on each parameter. The value at baseline (before treatment) was considered a covariate. All *p* values were adjusted according to the Tukey–Kramer multiple group comparison procedure. For time-point analysis, the paired t-test was applied. Significance was accepted as *p* < 0.05. Data are presented as LSMs ± SEs (*n* = 19); iFGF23, intact fibroblast growth factor; PTH, parathyroid hormone.

As we found differences in the quantity of calcium and phosphate in both types of protein isolates, we analyzed the postprandial levels of calcium, inorganic phosphate, as well as PTH and iFGF23, as these factors are important regulators of mineral homeostasis. Here, we found no differences in the postprandial levels of calcium, phosphate, PTH, and iFGF23 between the three treatments (Figure 6A–D). Likewise, the iAUC of calcium, phosphate, and iFGF did not differ among the three treatments (Table S2). However, the iAUC of PTH showed higher values after the consumption of TM+SPI than after the intake of TM+RPI or TM.

Since both types of plant proteins contained relatively high quantities of phytic acid, we measured the postprandial zinc and copper concentrations and assessed the iAUCs of zinc and copper. The postprandial zinc levels were moderately lower after the consumption of TM+RPI and TM+SPI than after the ingestion of TM, although this effect was not statistically significant (*p* = 0.09 and *p* = 0.11, respectively, Figure 6E). When assessing the iAUC of zinc, no differences were found between the three treatments (Table S2). Postprandial copper was not differentially influenced by the three treatments (Figure 6F).

### 3.8. Postprandial Urea and Amino Acids

**Figure 7:**
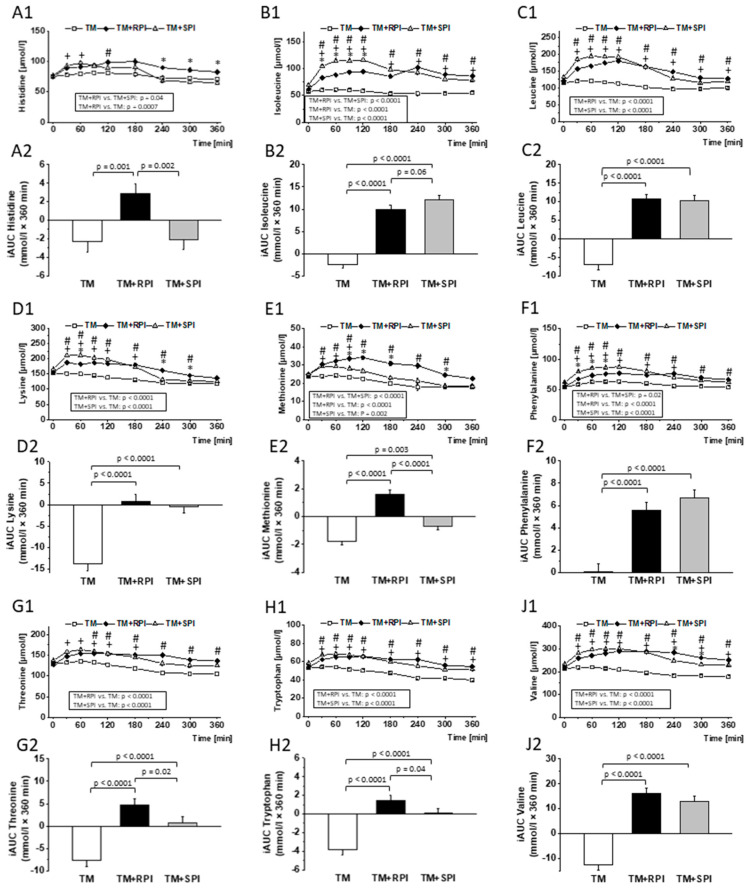
Postprandial response of the test meal (TM) with either no additional protein as reference (□), 28 g rapeseed protein isolate (RPI) (♦), or 28 g soy protein isolate (SPI) (▲) with regard to plasma levels and incremental area under the curve (iAUC) of (**A1**,**A2**) histidine, (**B1**,**B2**) isoleucine, (**C1**,**C2**) leucine, (**D1**,**D2**) lysine, (**E1**,**E2**) methionine, (**F1**,**F2**) phenylalanine, (**G1**,**G2**)threonine, (**H1**,**H2**) tryptophan, and (**J1**,**J2**) valine. Differences in amino acid concentrations after the ingestion of the three test meals over 6 h were evaluated with a mixed model to test the effects of treatment, time, and their interaction (treatment × time) on each parameter. The value at baseline (before treatment) was considered a covariate. The iAUC was calculated for each subject and treatment using the trapezoidal rule. The mixed-models procedure (PROC MIXED) was used for all traits. For iAUC, treatment, sex, sequence, and period were set as fixed effects, and subject was included as a random effect. All *p* values were adjusted according to the Tukey–Kramer multiple group comparison procedure. For time-point analysis, the paired t-test was applied. Significance was accepted as *p* < 0.05. Data are presented as LSMs ± SEs (*n* = 19). # *p* < 0.05 TM vs. TM+RPI; + *p* < 0.05 TM vs. TM+SPI; * *p* < 0.05 TM+RPI vs. TM+SPI.

Finally, to investigate the availability of RPI in comparison to that of SPI, we analyzed the postprandial concentrations of plasma amino acids and urea. The data show that the postprandial levels and iAUCs of indispensable amino acids, namely, histidine, isoleucine, leucine, lysine, methionine, phenylalanine, tryptophan, threonine, and valine, were higher after the intake of TM+RPI and TM+SPI, respectively, then after the consumption of TM without additional protein (Figure 7). Differences between TM+RPI and TM+SPI were observed for the postprandial levels of histidine, isoleucine, methionine, and phenylalanine. Treatment with TM+RPI was characterized by higher postprandial levels of histidine and methionine than treatment with TM+SPI (Figure 7A,E). In contrast, postprandial levels of isoleucine and phenylalanine were lower after the intake of TM+RPI than after that of TM+SPI (Figure 7B,F).

In addition, for the indispensable amino acids, the postprandial levels of most dispensable amino acids were higher after the intake of TM+RPI and TM+SPI than after the intake of TM without additional protein. However, the postprandial plasma levels of glutamic acid did not show differences between the three interventions. (Figure S3D). The iAUCs and confidence intervals of plasma amino acids after the consumption of the three test meals are presented in Table S3.

**Figure 8:**
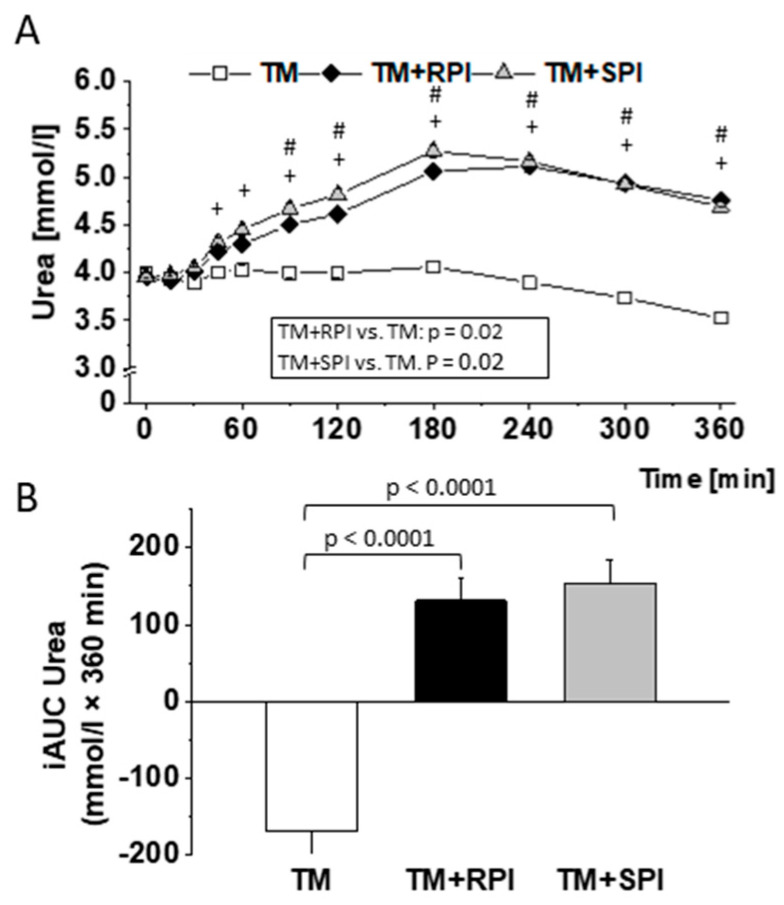
Postprandial response of the test meal (TM) with either no additional protein as reference (□), 28 g rapeseed protein isolate (RPI) (♦), or 28 g soy protein isolate (SPI) (▲) with regard to plasma levels (**A**) and incremental area under the curve (iAUC) (**B**) of urea. Differences in urea concentrations after the ingestion of the three test meals over 6 h were evaluated with a mixed model to test the effects of treatment, time, and their interaction (treatment × time) on each parameter. The value at baseline (before treatment) was considered a covariate. The iAUC was calculated for each subject and treatment using the trapezoidal rule. The mixed-models procedure (PROC MIXED) was used for all traits. For the iAUC, treatment, sex, sequence, and period were set as fixed effects, and subject was included as a random effect. All *p* values were adjusted according to the Tukey–Kramer multiple group comparison procedure. For time-point analysis, the paired t-test was applied. Significance was accepted as *p* < 0.05. Data are presented as LSMs ± SEs (*n* = 19). # *p* < 0.05 TM vs. TM+RPI; + *p* < 0.05 TM vs. TM+SPI.

The consumption of TM+RPI and TM+SPI resulted in a marked postprandial increase of urea compared to TM (Figure 8). The urea levels did not reach the baseline levels within 6 h postprandial. No differences in postprandial urea was observed between TM+RPI and TM+SPI.

## 4. Discussion

Due to a growing world population and the demand for sustainable food production, plant-based protein sources have become increasingly important in human nutrition. The vital aspects of using plant proteins as food components are the quantities of indispensable amino acids, biofunctional effects, and safety. A promising protein source is rapeseed, as it is characterized by an amino acid profile comparable to that of soy. The health and safety aspects of novel protein sources require attention from researchers. Studies on postprandial metabolism are an excellent way to obtain a comprehensive view of the metabolic impact, safety, and bioavailability of novel food components.

Here, we investigated the impact of rapeseed protein isolate in comparison to soy protein isolate on the postprandial concentrations of metabolites and hormones in healthy subjects who received a test meal rich in carbohydrates and fat. To our knowledge, this is the first human study that examines the impact of rapeseed protein on the postprandial response of glucose metabolism, plasma lipids, satiety, and other health relevant factors. Thus, the study provides valuable data that are necessary to assess the potential of rapeseed protein as a food ingredient. Overall, we found that rapeseed protein isolate and soy protein isolate, which were administered together with the test meal, resulted in a series of comparable postprandial responses. These include responses in blood pressure, postprandial lipids, minerals, and urea. Interestingly, significant differences between the two protein isolates were found for satiety, postprandial glucose metabolism, and a few dispensable and indispensable amino acids.

Dietary proteins largely differ in their quantity of indispensable amino acids, and plant proteins are normally characterized by a lower content of indispensable amino acids than animal proteins [46]. The current data show that rapeseed protein has quantities of indispensable and dispensable amino acids comparable to those of soy protein, which is considered to meet the requirements recommended by the WHO/FAO [47]. It is assumed that postprandial amino acids reflect the digestibility of proteins [48]; it must be considered that absorbed amino acids first pass the liver before use, e.g., for protein synthesis or degradation. In addition, plasma amino acids may also derive directly from the tissue turnover and protein degradation [49]. Nonetheless, postprandial amino acids are of particular interest because they appear to influence satiety [50] and glucose and insulin metabolism [51]. Despite the favorable amino acid profile of both proteins, we observed a marked postprandial increase in urea after consumption of the protein-enriched test meals. We assume that the increase in urea was caused by the large quantity of ingested protein, not by the poor protein quality. Together with the TM that provided 16 g protein, the subjects treated with TM+RPI or TM+SPI consumed 41 g protein. Data from intervention studies with athletes showed that 20 g of whey protein was the maximum quantity of protein that can be utilized in healthy men after exercise and in rest, whereas protein consumption above this level results in urea production [52,53,54]. However, it should be considered that individual factors (e.g., muscle mass, height, body weight and age) and the type of ingested protein determine the maximum amount of utilized protein. Thus, it can be assumed that there are also subjects who can utilize more than 20 g of protein. The marked increase in postprandial urea after the consumption of the proteins which are indicative of an increased degradation of amino acids that exceeded those required, calling into question the suitability of a high-protein diet.

Importantly, participants reported a stronger feeling of satiety after the consumption of the test meal with rapeseed protein than after the intake of the test meal alone. At first view, the satiating effect of rapeseed protein appears plausible because intake of an additional 28 g protein isolate provided approximately 100 kcal. However, this effect was not observed when soy protein isolate was added to the test meal. Thus, we conclude that rapeseed protein has a distinct satiating effect compared to soy protein. Protein-induced satiety has been demonstrated in a few studies [55,56,57], and the magnitude of satiety has been shown to differ between various proteins [58,59]. Veldhorst et al. were able to demonstrate that protein-derived amino acids exert differential effects on satiety [58]. It is assumed that single amino acids may be responsible for the protein-specific effect on satiety because studies found an increased intake of leucine, lysine, tryptophan, isoleucine, and threonine, which was accompanied by an increased plasma concentration of these amino acids and was associated with a reduced feeling of hunger [58]. The observed differences in the plasma levels of tryptophan and threonine in subjects consuming rapeseed and soy protein may possibly explain the satiating effect of rapeseed protein, although it must be clearly stated that the study was not designed to provide mechanistic explanations.

Next, we investigated the influence of rapeseed and soy protein on the cardiovascular risk factors glucose and insulin, lipids, and the inflammatory marker IL-6. Interestingly, rapeseed protein and soy protein exert differential effects on postmeal glucose metabolism, particularly insulin. Compared with the test meal alone, rapeseed protein, but not soy protein, resulted in lower postprandial levels of glucose. Additionally, we found that the insulin response was higher after the consumption of soy protein than after that of rapeseed protein. The glycemic impact of dietary proteins is still a matter of debate. There is consensus that both the amount and type of ingested protein can modulate glucose homeostasis. For example, Kashima et al. found that the ingestion of 40 g versus 20 g soy protein can lower postprandial blood glucose [3]. Similar results were found by Akhavan et al. [60], who administered different quantities of whey protein. Regarding the protein type, Schopen et al. demonstrated that lupin protein, which was added to a carbohydrate-rich reference meal, can lower the postprandial increase in blood glucose in comparison to a meal with whey protein [36]. One mechanism that may explain the differential effects of dietary proteins on glucose includes the modulation of insulin secretion by specific amino acids [61]. In the current study, the postprandial plasma amino acid patterns were comparable between the two protein interventions, suggesting that the amino acids were probably not responsible for the observed effect on blood glucose. However, the reduction of glucose levels can also be caused by insulin-independent mechanisms such as gastric emptying. Data indicate that dietary proteins are capable of modulating gastric emptying [62]. Since rapeseed protein isolate does not only induce a reduction in postprandial glucose concentration but induces also a greater satiety after its ingestion, we hypothesize that both proteins may have affected differently the gastric emptying rate. However, this hypothesis would have to be examined in further studies.

As high postprandial glucose and insulin levels have been associated with an increased risk of cardiovascular diseases in healthy individuals and in patients with diabetes [63,64,65,66], we conclude that rapeseed protein had a more favorable health impact on postprandial glucose metabolism than soy protein.

Another postprandial metabolite that has been considered to be an independent risk factor for atherosclerosis is triglycerides [67,68], and data indicate differential effects of various protein sources and plasma amino acids on postprandial lipids [69,70]. Here, we found that the addition of the two proteins resulted in higher postprandial levels of triglycerides and cholesterol than the test meal alone. Increased plasma levels of isoleucine, leucine, valine, and phenylalanine were found to be associated with hypertriglyceridemia [71,72]. Thus, we assume that the changes in postprandial lipids could be caused by the increase in circulating amino acids.

The cytokine IL-6 is a key mediator of inflammation [73] and is suggested to be linked to an increased risk of cardiovascular events [74]. In line with previous reports [70,73,75], we observed a marked postprandial increase in IL-6 after all treatments. Based on data from several studies, it is suggested that the postprandial rise in IL-6 is caused by dietary fats, which can stimulate the Toll-like receptor 4 pathway [76,77,78]. Considering the reference value for IL-6, which has been set to < 11.3 pg/mL [79], our data indicate a pro-inflammatory postprandial condition in response to all experimental meals applied. The current data are not indicative of a modulating effect of plant proteins on the postprandial inflammatory response because the postprandial IL-6 curves were comparable between the three treatments.

Plant protein isolates often contain phytic acid, which is an anti-nutritive factor that can deteriorate the uptake of minerals, such as zinc [24]. Analysis of the currently used protein isolates revealed relatively high quantities of phytic acid, 2 g/100 g, in both types of protein isolates. On the other hand, protein isolates are also sources of minerals. The main differences in minerals between the two protein isolates used in our study were observed in calcium, with considerably higher amounts in the rapeseed protein isolate than in the soy protein isolate. Dietary calcium is suggested to have beneficial effects on e.g., blood pressure and hypertensive disorders, cardiovascular mortality, and colorectal cancer [80,81,82]. However, data published so far are not indicative of any calcium effect on satiety and glucose metabolism [83,84], which we identified as the most relevant parameters that differ between the two proteins. However, these differences in minerals between the three treatment meals were not associated with differences in the postprandial levels of calcium, inorganic phosphate, and their regulating hormone iFGF23. PTH was elevated after the ingestion of TM+SPI but not after TM+RPI compared to the levels following TM consumption. It is possible to speculate that the differential effects on PTH were caused by the differences in phosphate and calcium contents of the proteins because the soy protein contained higher quantities of inorganic phosphate and lower quantities of calcium than rapeseed protein [85]. To elucidate a possible impact of phytic acid on cationic trace element absorption, we analyzed postprandial zinc and found a trend toward lower postprandial zinc levels after the consumption of the test meals with rapeseed or soy protein (*p* = 0.09 and *p* = 0.11, respectively), although both protein-enriched test meals contained 15–20% more zinc than the test meal alone. Thus, we speculate that phytic acid-rich plant protein isolates can slightly reduce the bioavailability of trace elements.

### Strengths and Limitations of the Study

The strength of our study was that we used protein isolates with a high and comparable protein content that contained only small quantities of nonprotein compounds, which could have influenced the postprandial metabolites. Additionally, the study was conducted as a double-blind, controlled trial in a crossover design, in which the interventions were compared within the same individuals to eliminate between-subject variability. Since the sequence of the three treatments and the period had no influence on the outcome of this study, we can exclude carry-over effects. However, data interpretation is restricted to the postprandial effects of the proteins. No conclusions can be drawn concerning the long-term effects of the treatments with rapeseed protein.

In conclusion, both rapeseed and soy protein provide a favorable amino acid composition for human health. These proteins were also comparable concerning the postprandial responses induced with regard to blood pressure, lipids, minerals, and urea. Interestingly, the rapeseed protein had a stronger effect on satiety than the soy protein. Additionally, we found a more favorable impact of the rapeseed protein on postprandial glucose metabolism than that of the soy protein. These findings indicate that rapeseed protein has value as a food component.

## Figures and Tables

**Table 1:** Energy and quantities of macronutrients and minerals in the three test meals.

	Test Meal	Test Meal + 28 g RPI	Test Meal + 28 g SPI
Calculated			
Energy [kcal]	693	808	802
Protein [g]	16	41	41
Carbohydrates [g]	93	93	93
Fat [g]	23.5	25.2	24.5
Analyzed			
Na [mg]	519	681	832
K [mg]	489	500	544
Ca [mg]	92	193	80
P [mg]	234	364	439
Zn [mg]	2.2	2.6	2.8
Cu [mg]	0.6	6.2	0.9

RPI, rapeseed protein isolate; SPI, soy protein isolate; Analyses were run in duplicate.

**Table 2:** Composition of the protein isolates determined by analyses.

	Rapeseed Protein Isolate	Soy Protein Isolate
Dry Matter (DM) [%]	93.6	94.1
g/100 g DM		
Crude protein	92.6	90.6
Crude fat	6.08	3.71
Crude ash	4.32	4.68
Phytic acid	1.97	1.96
**Amino Acids**		
Alanine	3.50	3.86
Arginine	5.64	7.00
Aspartate	5.55	10.3
Cysteine	2.68	1.05
Glutamate	20.6	18.0
Serine	3.46	4.60
Glycine	4.21	3.72
Histidine	3.11	2.81
Isoleucine	2.94	4.00
Leucine	6.49	7.36
Lysine	4.87	5.58
Methionine	1.82	1.21
Phenylalanine	3.17	4.49
Proline	6.22	4.44
Threonine	3.14	3.31
Tryptophan	1.01	0.98
Tyrosine	1.26	3.34
Valine	3.72	3.81

Analyses were run in duplicate.

**Table 3:** Quantities of minerals in the protein isolates determined by analyses.

	Rapeseed Isolate	Soy Protein Isolate
mg/100 g DM		
Sodium	650	980
Potassium	100	230
Calcium	490	50
Phosphorus	640	910
Zinc	2.1	2.8
Copper	22.1	1.2

Analyses were run in duplicate. DM, dry matter.

**Table 4:** Characteristics of study participants at baseline.

Characteristics	
Age [years]	31 ± 3 (18, 64)
Male/female [%]	25/75
Body weight [kg]	68.4 ± 3.1 (50, 108)
Body mass index [kg/m^2^]	23.8 ± 0.7 (18.6, 30.1)
Blood pressure [mmHg]	
Systolic	113 ± 4 (91, 156)
Diastolic	77 ± 2 (64, 99)
Heart rate [beats/min]	78 ± 2 (59, 94)
Glucose [mmol/L]	4.9 ± 0.1 (4.2, 5.45)
Insulin [mIU/L]	14.4 ± 14.0 (5.5, 25.9)
Triglycerides [mmol/L]	0.9 ± 0.1 (0.5, 2.6)
Cholesterol [mmol/L]	4.2 ± 0.3 (0.5, 6.4)
hsCRP [µg/mL]	1.36 ± 0.40 (0.15, 6.52)
IL-6 [pg/mL]	0.9 ± 0.1 (0.1, 9.0)
iFGF23 [pg/mL]	43.9 ± 3.1 (25.0, 75.1)
PTH [pg/mL]	30.4 ± 5.2 (11.4, 81.0)
Inorganic phosphate [mmol/L]	1.19 ± 0.04 (0.8, 1.6)
Calcium [mmol/L]	2.36 ± 0.06 (1.69, 2.65)
Zinc [µg/L]	841 ± 53 (501, 1467)
Copper [µg/L]	1158 ± 91 (658, 2321)
Urea [mmol/L]	4.2 ± 0.2 (2.1, 5.9)

Values are given as the means ± SEMs (min; max), *n* = 20; hs-CRP, highly sensitive C-reactive protein; iFGF23, intact fibroblast growth factor 23; IL-6, interleukin 6; PTH, parathyroid hormone.

## Supplementary Materials

The following are available online at https://www.mdpi.com/2072-6643/12/8/2270/s1, Figure S1: Design of the crossover study, Figure S2: Postprandial blood pressure and heart rate, Figure S3: Postprandial dispensable amino acid response, Table S1: Baseline amino acid concentrations of the study participants, Table S2: iAUC and confidence intervals for plasma parameters. Table S3: iAUCs and confidence intervals of plasma amino acids.

## Abbreviations

BMI	body mass index
FAO	Food and Agriculture Organization of the United Nations
FDA	Food and Drug Administration
iFGF23	intact fibroblast growth factor 23
hs-CRP	high-sensitivity C-reactive protein
iAUC	incremental area under the curve
IL-6	interleukin-6
LSM	least square mean
PTH	parathyroid hormone
RPI	rapeseed protein isolate
SPI	soy protein isolate
TM	test meal
VAS	visual analog scale
WHO	World Health Organization
