# Supplementary material for: Postprandial Metabolic Response to Rapeseed Protein in Healthy Subjects
Source: Nutrients. 2020 Jul 29;12(8):2270. doi: 10.3390/nu12082270 (PMC7469072; doi:10.3390/nu12082270)
Supplement: Supplementary file 1 [file nutrients-12-02270-s001.pdf]

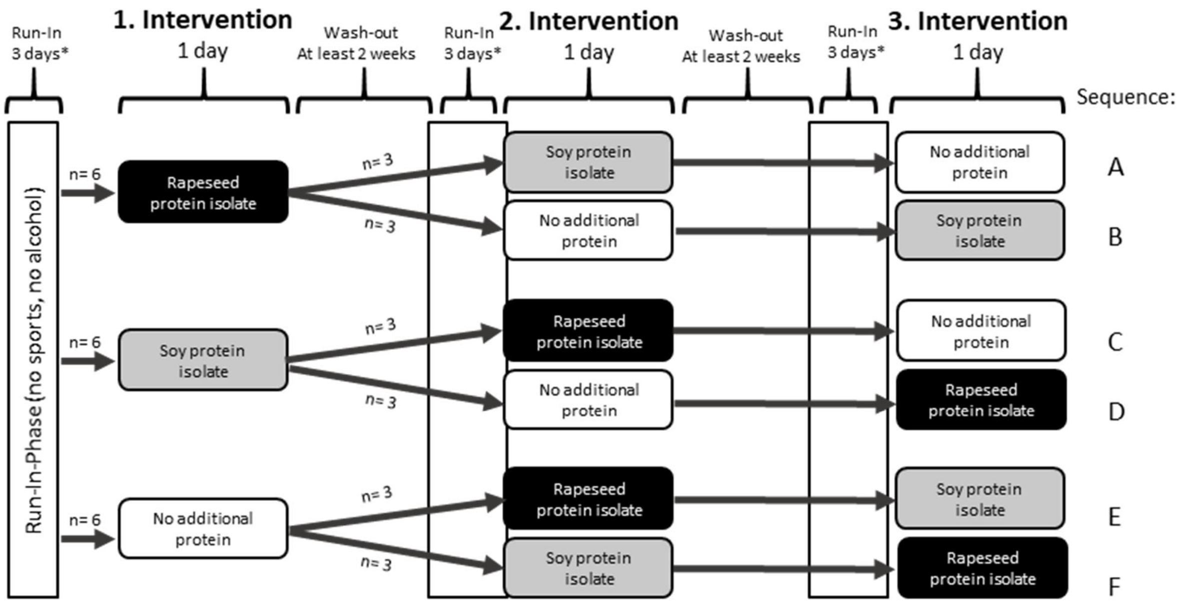

Figure S1 Design of the crossover study in which participants received test meals with rapeseed protein, soy protein or no protein in random order
*Standardized dinner the evening prior to intervention

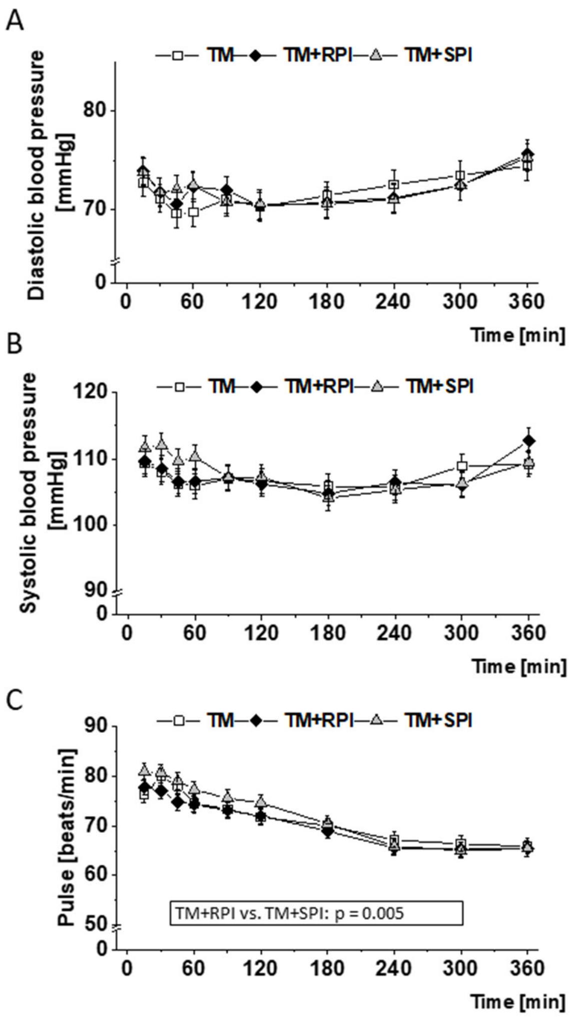

Figure S2: Postprandial (A) diastolic blood pressure, (B) systolic blood pressure and (C) pulse of study participants who received either the test meal (TM) without additional protein (□), the TM with 28 g rapeseed protein isolate (RPI) (♦) or the TM with 28 g soy protein isolate (SPI) (▲). Differences in blood pressure and pulse after the ingestion of the three test meals over 6 h were evaluated with a mixed model to test the effects of treatment, time, and their interaction (treatment × time) on each parameter. The value at baseline (before treatment) was considered a covariate. All p values were adjusted according to the Tukey-Kramer multiple group comparison procedure. For time point analysis, the paired t test was applied. Significance was accepted as $p < 0.05$. Data are presented as LSMs ± SEs (n = 19).

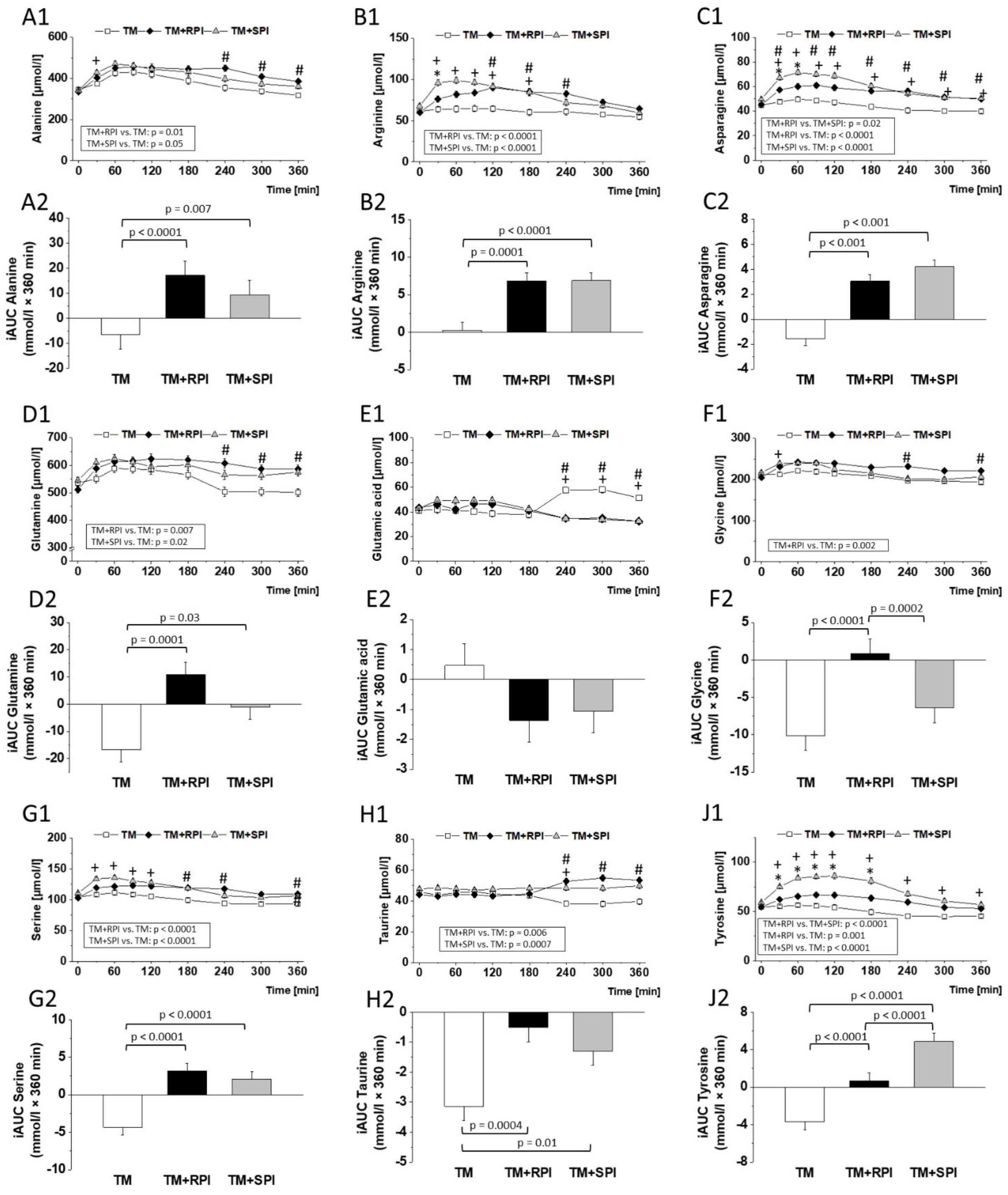

Figure S3 Postprandial response of the test meal (TM) with either no additional protein as reference (□), 28 g rapeseed protein isolate (RPI) (♦) or 28 g soy protein isolate (SPI) (▲) with regard to plasma levels and the incremental area under the curve (iAUC) of (A1 + 2) alanine, (B1 + 2) arginine, (C1 + 2) asparagine, (D1 + 2) glutamine, (E1 + 2) glutamic acid, (F1 + 2) glycine, (G1 + 2) serine, (H1 + 2) taurine and (J1 + 2) tyrosine. Differences in amino acid concentrations after the ingestion of the three test meals over 6 h were evaluated with a mixed model to test the effects of treatment, time, and their interaction (treatment × time) on each parameter. The value at baseline (before treatment) was considered a covariate. The iAUC was calculated for each subject and treatment using the trapezoidal rule. The mixed-models procedure (PROC MIXED) was used for all traits. For iAUC, treatment, sex, sequence

and period were set as fixed effects, and subject was included as a random effect. All p values were adjusted according to the Tukey-Kramer multiple group comparison procedure. For time point analysis, the paired t test was applied. Significance was accepted as $p < 0.05$. Data are presented as LSMs ± SEs ($n = 19$); $^{\#}$ $p < 0.05$ TM vs. TM+RPI; $^{+}$ $p < 0.05$ TM vs. TM+SPI; * $p < 0.05$ TM+RPI vs. TM+SPI.

Table S1. Baseline amino acid concentrations of the study participants

**Amino acid [µmol/l]**	
Alanine	386 ± 14.9 (315; 557)
Arginine	61 ± 4.6 (26; 100)
Asparagine	51 ± 1.8 (35; 68)
Glutamine	602 ± 22.5 (395; 766)
Glutamic acid	44 ± 4.1 (22; 96)
Glycine	237 ± 15.1 (131; 403)
Histidine	91 ± 3.6 (73; 130)
Isoleucine	62 ± 3.0 (40; 92)
Leucine	126 ± 6.0 (79; 184)
Lysine	168 ± 6.3 (114; 233)
Methionine	26 ± 0.9 (20; 33)
Phenylalanine	60 ± 1.6 (49; 74)
Serine	115 ± 4.876; 155)
Taurine	54 ± 2.5 (33;75)
Threonine	138 ± 9.1 (77; 231)
Tryptophan	58 ± 1.9 (41; 76)
Tyrosine	60 ± 3.1 (36;81)
Valine	231 ± 8.4 (183; 319)

Values are given as mean ± SEM (min, max).

Table S2 iAUC and confidence intervals for plasma parameters after consumption of the three test meals

iAUC	TM	95% CI	TM+RPI	95% CI	TM+SPI	95% CI
Glucose [mmol/l × 180 min]	103	59.2; 147	**52.0†**	**8.03; 96.0**	79.7	35.7; 123
Insulin [IU/l × 180 min]	4.88	3.93; 5.83	5.42	4.47; 6.39	**6.42***	**5.47; 7.37**
Triglycerides [mmol/l × 360 min]	-25.8	-48.7; -2.85	**6.00***	**-16.9; 28.9**	**-0.13***	**-23.0; 22.8**
Cholesterol [mmol/l × 360 min]	-241	-280; -203	-214	-252; -175	-193	-231; -155
Urea [mmol/l × 360 min]	-168	-230; -107	**132***	**70.2; 193**	**154***	92.9; 215
Phosphate [mmol/l × 360 min]	-62.2	-82.8; -41.7	-65.6	-86.1; -45.1	-51.7	-72.2; -31.2
PTH [ng/ml × 360 min]	-1.72	-3.80; 0.37	-1.16	-3.25; 0.92	**-0.07***	**-2.15; 2.02**
FGF23 [ng/ml × 360 min]	-2.13	-2.94; -1.33	-1.76	-2.56; -0.96	-1.82	-2.63; -1.02
Calcium [mmol/l × 360 min]	-88.3	-120; -57.0	-94.8	-126; -63.5	-78.5	-110; -47.3
Zinc [mg/l × 360 min]	-71.8	-76.8; -23.0	-74.3	-85.4; -2.0	-58.5	-84.6; -32.4
Copper [mg/l × 360 min]	-31.9	2.49; 66.0	-34.2	-63.8; -0.12	-4.11	-27.6; 35.9
IL-6 [ng/ml × 360 min]	1.20	0.54; 1.96	1.32	0.61; 2.15	2.20	0.58; 2.05

Values are LSMs, n = 19; * Different from TM, $p < 0.05$; † $p = 0.06$;

The incremental area under the curve (iAUC) was calculated for each subject and treatment using the trapezoidal rule. The mixed-models procedure (PROC MIXED) was used for all traits. For the iAUC treatment, sex, sequence and period were set as fixed effects, and subject was included as a random effect. All p values were adjusted according to the Tukey-Kramer multiple group comparison procedure.

.

Table S3 iAUCs and confidence intervals of plasma amino acids after the consumption of the three test meals

iAUC	TM	95% CI	TM+RPI	95% CI	TM+SPI	95% CI
Alanine	-6.08	-18.5; 5.33	**17.9***	**5.27; 29.1**	**10.2***	**-2.50; 21.3**
Arginine	0.31	-1.85; 2.36	**7.08***	**4.77; 8.97**	**7.11***	**4.80; 9.00**
Asparagine	-1.59	-2.63; -0.53	**2.99***	**1.97; 4.11**	**4.21***	**3.15; 5.25**
Glutamine	-17.1	-25.6; -7.90	**10.6***	**2.10; 19.8**	**-1.52***	**-10.0; 7.66**
Glutamic acid	0.52	-0.99; 1.94	-1.30	-2.86; 0.13	-1.06	-2.53; 0.40
Glycine	-9.99	-13.3; -6.62	**0.88*°**	**-1.01; 5.86**	**-6.36**	**-9.80; -3.09**
Histidine	-2.34	-4.52; -0.15	**2.88*°**	**0.70; 5.05**	**-2.09**	**-4.27; 0.09**
Isoleucine	-2.41	-4.34; -0.48	**9.97***	**8.05; 11.9**	**12.1***	**10.1; 14.0**
Leucine	-6.98	-9.75; -4.22	**10.7***	**7.89; 1.34**	**10.3***	**7.50; 1.30**
Lysine	-13.8	-17.0; -10.7	**0.84***	**-2.40; 4.07**	**-0.51***	**-3.66; 2.64**
Methionine	-1.79	-2.35; -1.23	**1.61*°**	**0.90; 2.31**	**-0.69***	**-1.31; -0.06**
Phenylalanine	0.09	-1.39; 1.56	**5.56***	**4.09; 7.03**	**6.68***	**5.21; 8.15**
Serine	-4.28	-6.43; -2.26	**3.09***	**1.08; 5.24**	**2.03***	**0.01; 4.18**
Taurine	-3.14	-4.09; -2.20	**-0.63***	**-1.46; 0.43**	**-1.41***	**-2.25; -0.36**
Threonine	-7.71	-10.0; -5.22	**4.65*°**	**3.24; 8.17**	**0.81***	**-1.60; 3.18**
Tryptophan	-3.85	-4.99; -2.72	**1.44*°**	**0.30; 2.57**	**0.06***	**-1.08; 1.19**
Tyrosine	-3.54	-5.43; -1.94	**0.83*°**	**-1.08; 2.46**	**4.78***	**3.10; 6.63**
Valine	-12.7	-16.8; -8.54	**16.3***	**12.1; 20.4**	**12.9***	**8.78; 17.0**

Values are LSMs, n = 19; * Different from TM, $p < 0.05$; ° Different from TM+SPI, $p < 0.05$

The incremental area under the curve (iAUC) was calculated for each subject and treatment using the trapezoidal rule. The mixed-models procedure (PROC MIXED) was used for all traits. For iAUC treatment, sex, sequence and period were set as fixed effects, and subject was included as a random effect. All p values were adjusted according to the Tukey-Kramer multiple group comparison procedure.

CI, confidence interval
